# New Target Therapies in Advanced Non-Small Cell Lung Cancer: A Review of the Literature and Future Perspectives

**DOI:** 10.3390/jcm9113543

**Published:** 2020-11-03

**Authors:** Ramon Andrade de Mello, Nathália Moisés Neves, Hakaru Tadokoro, Giovanna Araújo Amaral, Pedro Castelo-Branco, Victor André de Almeida Zia

**Affiliations:** 1 Algarve Biomedical Centre, Department of Biomedical Sciences and Medicine, University of Algarve (DCBM UALG), 8005-139 Faro, Portugal; htadokoro@uol.com.br (H.T.); castelobranco.pedro@gmail.com (P.C.-B.); 2 Division of Medical Oncology, Escola Paulista de Medicina, Federal University of São Paulo (UNIFESP), São Paulo 04037-004, SP, Brazil; nathalia.neves@unifesp.br (N.M.N.); gioaraujoamaral@gmail.com (G.A.A.); victor_zia_oncologia@outlook.com (V.A.d.A.Z.); 3 Precision Oncology and Health Economics Group (ONCOPRECH), Post-Graduation Program in Medicine, Nine of July University (UNINOVE), São Paulo 04037-004, SP, Brazil; 4 Division of Oncology, Algarve Biomedical Centre, Department of Biomedical Sciences and Medicine, University of Algarve, 8005-139 Faro, Portugal

**Keywords:** non-small-cell lung cancer, target therapy, tyrosine kinase inhibitors, ALK inhibitors, immunotherapy

## Abstract

Introduction: Lung cancer (LC) is the most common neoplasm worldwide, and 85% of these tumors are classified as non-small cell lung cancer (NSCLC). LC treatment was initially restricted to cytotoxic chemotherapy—platinum compounds associated with 3rd generation cytotoxic agents (paclitaxel, gemcitabine, pemetrexed) and, more recently, with monoclonal antibodies (bevacizumab, ramucirumab). Advancements in treatment are correlated with prolonged overall survival (OS). Current advances are focused on target therapies. Target agents: Anti-epidermal growth factor receptor (EGFR) therapy consists of 1st and 2nd generation tyrosine kinase inhibitors (TKIs such as erlotinib, afatinib). In 60% of cases, resistance to these TKIs occurs due to T790M mutation in EGFR, which is overcome 3rd generation drugs (osimertinib). Anaplastic lymphoma kinase (ALK) is the target for drugs such as crizotinib, alectinib, ceritinib. Programmed death 1 (PD-1) and its ligand serve as targets for immunotherapy agents such as pembrolizumab, nivolumab, atezolizumab. Discussion: Challenges in NSCLC treatment include resistance to 3rd generation TKIs, the high cost of ALK inhibitors, and the need for further research on new drugs.

## 1. Introduction

### 1.1. Epidemiology

Lung cancer (LC) is the most common neoplasm worldwide [1]. The World Health Organization (WHO) reported 2.1 million new cases of LC in 2018, with the highest incidence rates in Turkey, Japan, China, the United States, and the United Kingdom [2]. Specifically, in the United States, the American Cancer Society reports that LC was the malignancy with the second-highest number of estimated new cases in 2019, both in men and women. Lung and bronchus cancer had an estimated 228,150 new cases in the United States during 2019, divided between men (116,440 cases) and women (111,710 cases) [3]. The term “lung cancer” accounts for a heterogeneous set of tumors. They are divided into non-small cell lung cancer (NSCLC) and small cell lung cancer (SCLC). Among them there are more than 50 different histological subtypes [4] NSCLC, for instance, is subcategorized into adenocarcinoma, squamous cell carcinoma, and large cell carcinoma [5]. In this review of the literature, we will focus on NSCLC, which accounts for around 85% of all LC cases [6].

### 1.2. Mortality

LC is the main cause of death worldwide and accounts for approximately 1.6 million deaths per year [7]. In 2018, the WHO reported 1.8 million deaths due to this malignancy. The highest LC mortality rates were seen in countries such as Turkey, Canada, United States, United Kingdom, and Japan [2]. The American Cancer Society estimates that, in 2019, LC was the most important cause of cancer-related deaths in American men and women. During that year, lung and bronchus cancer had an estimated 76,650 male deaths and 66,020 female deaths, totaling 142,670 deaths in the United States. For all stages combined, LC has a 19% five-year relative survival rate [3]. The high mortality of this malignancy is heightened by the fact that many tumors are already advanced at the moment of diagnosis. Studies have found the presence of distant-organ metastasis in 47.3% of patients with NSCLC at their initial cancer diagnosis, which can greatly hinder treatment [8]. Therefore, mortality is still a very important problem related to this malignancy, and novel therapies aim to reduce its rates.

### 1.3. Risk Factors

The main risk factor for developing LC is tobacco smoking, which is accounted for in at least 80% of cases. Continuous smoking can increase LC risk by up to 50%, and in these cases, the association with squamous-cell LC is stronger than other tumor subtypes [7]. Other important environmental risk factors are passive smoking (regular passive smoking can increase LC risk up to 30%), pollution (a European cohort found that exposure to particulate matter is associated with a hazard ratio of 1.22 for LC), and occupational exposure to carcinogens (such as asbestos, chromium compounds, silica and diesel fumes) [9,10]. Furthermore, studies have shown that having a first-degree relative with a history of LC increases a person’s risk of developing this disease by 50% and that around 8% of all LC cases occur due to genetic predisposition [11].

### 1.4. Evolution in Treatment

Considerable advances have been made regarding LC treatment, which was initially restricted to cytotoxic chemotherapy [12]. Chemotherapy agents block the cell cycle and therefore attack rapidly dividing cells. This traditional treatment has low specificity, attacking normal cells as well as cancerous ones, leading to significant side effects. The recent advances in cancer treatment consist of the development of target therapies, which are drugs that block specific molecules that are found over-expressed or overactive in tumor cells only. These altered target molecules are a result of driver mutations in the cancer cells, which amplify proliferation and survival pathways, leading to tumor growth. There are roughly three types of target therapies: monoclonal antibodies, small molecule inhibitors, and immunotoxins [13,14]. The impact of this evolution of LC treatment is reflected in the increase of 5-year survival rates of patients, which jumped from 10.7% in the early 1970s to 19.8% in the 2010s [15]. However, some challenges remain, such as the identification of new driver gene alterations involved in carcinogenesis, knowledge of drug-resistance mechanisms, and recognition of response predictors to novel therapies [7].

### 1.5. Advanced Disease: From Cytotoxic Therapy to Target Agents

#### 1.5.1. Cytotoxic Therapy

Chemotherapy was the backbone of lung cancer treatment for several years. Before the chemotherapy era, the median overall survival (OS) of metastatic lung cancer was 3.9 months with the best supportive care [16]. Platinum combinations have been playing the main role in treatment since the 80s, but the landmark study of these drugs was the meta-analysis by the Non-Small Cell Lung Cancer Collaborative Group in 1995, which demonstrated that platinum-based chemotherapy significantly improved OS over best supportive care (15% vs. 5% OS rate in 1 year) [17]. This method of chemotherapy is still prescribed to up to 80% of patients diagnosed with lung cancer, whether isolated or combined with radiotherapy. This modality has been used in several different scenarios: adjuvant for patients with compromised lymph nodes, neoadjuvant to reduce the tumor mass in advanced stages, as part of palliative systemic care in patients with metastatic disease, or in patients who can’t undergo surgery, regardless of tumor staging [1]. Platinum-based compounds (cisplatin, carboplatin) have been associated with third-generation cytotoxic agents. This combination is referred to as “platinum doublets” and can have double the response rate (RR) when compared to monotherapy regimens [18]. When comparing the effects of different doublets, studies found similar response rates for paclitaxel (RR = 0.89) and gemcitabine (RR = 0.92) in the doublets, while docetaxel had a smaller response rate (RR = 0.76) [19].

During the 2000s, another player entered the game. In phase 3 JMDB trial, pemetrexed-cisplatin doublets demonstrated clinical benefit (increase in OS of 1.7 months) on non-squamous NSCLC when compared to gemcitabine-cisplatin doublets, supporting the concept that histology does matter in the treatment of lung cancer [17]. Another phase 3 trial demonstrated the clinical benefit of pemetrexed maintenance treatment in non-squamous NSCLC. Pemetrexed was the only cytotoxic agent found to improve both progression-free survival and OS in maintenance therapy, and it was also the drug that caused the least toxic adverse effects in patients [20].

**Figure 1:**
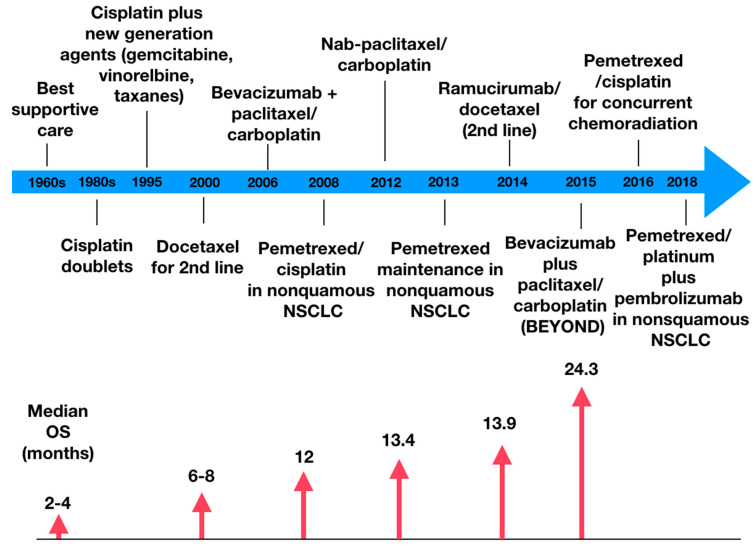
Evolution of chemotherapy treatment in non-small cell lung cancer, highlighting some landmark drugs and the accompanying increase in median overall survival. NSCLC = non-small cell lung cancer. OS = overall survival.

Figure 1 demonstrates the evolution of the clinical benefit of chemotherapy; as it becomes more precise, the higher is the overall survival [17].

#### 1.5.2. Chemotherapy with Other Agents

Bevacizumab is a monoclonal antibody that binds to vascular endothelial growth factor receptor (VEGFR) in cancer cells, blocking angiogenic pathways and therefore decreasing a tumor’s vascular permeability, which can decrease tumor growth from 25 to 95% [21]. There are several trials in which the efficacy of this drug has been proved. A meta-analysis demonstrated that the use of a platinum doublet plus bevacizumab showed significant OS benefit over platinum doublet alone, reducing mortality by 11% [22]. In the phase III ECOG 4599 trial, bevacizumab was combined with paclitaxel/carboplatin, leading to higher response rates and OS (12.3 months vs. 10.3 months, *p* = 0.003) when compared to paclitaxel/carboplatin alone [23]. Several other trials demonstrated the clinical benefit of bevacizumab, as the AVAil trial and the BEYOND trial [24]. Despite the benefits, this agent is associated with life-threatening bleeding and is only recommended for tumors or non-squamous histology [25]. Combinations with platinum doublets have been evaluated in the adjuvant setting but failed to prove OS benefit [26].

Ramucirumab is a human monoclonal antibody with a high affinity to the VEGFR2 extracellular domain. It can be used to treat patients with locally advanced or metastatic NSCLC [27]. This indication is supported by several studies, for instance, the REVEAL trial, a multicenter, double-blind, randomized phase III study that compared Ramucirumab plus Docetaxel versus placebo plus Docetaxel as a second-line treatment of NSCLC after disease progression on platinum-based chemotherapy. The results showed a higher median OS in the first group (10.5 months) compared to the second group (9.1 months). Median progression-free survival (PFS) has also been proven higher in the Ramucirumab group. However, almost all patients in both arms presented treatment-emergent adverse effects, and the common grade 3 adverse effects in the Ramucirumab group were neutropenia, leucopenia, fatigue, and hypertension [28].

Albumin-bound paclitaxel is a microtubule inhibitor best efficient when used to treat NSCLC, either as a single therapy or as part of some combination [29] Albumin-bound paclitaxel, also known as nab-Paclitaxel, is a solvent-free formulation developed over a decade ago that delivers a higher dose of Paclitaxel to solid tumors while reducing the incidence of toxicities [30]. Regarding NSCLC, a phase III trial shows that, based on independent assessment, weekly nab-Paclitaxel plus Carboplatin demonstrated a higher overall response rate (ORR, 33%) than solvent based-Paclitaxel plus Carboplatin (25%). The nab-Paclitaxel arm also presented a higher median OS (12.1 vs. 11.2 months) and median PFS (6.3 vs. 5.8 months) compared to the other group (*p* values = 0.271 and 0.214, respectively). Nab-paclitaxel has also shown benefit as the patients who received it presented significantly less grade 3 or higher adverse effects, such as neuropathy, neutropenia, arthralgia, and myalgia. However, the drug demonstrated a higher incidence of thrombocytopenia and anemia [31]. Current guidelines recommend nab-paclitaxel as a first-line replacement of docetaxel or paclitaxel for those who experience hypersensitivity reactions despite pre-medications or where pre-medications are contraindicated [32].

## 2. Target Agents

### 2.1. Anti-EGFR

**Figure 2:**
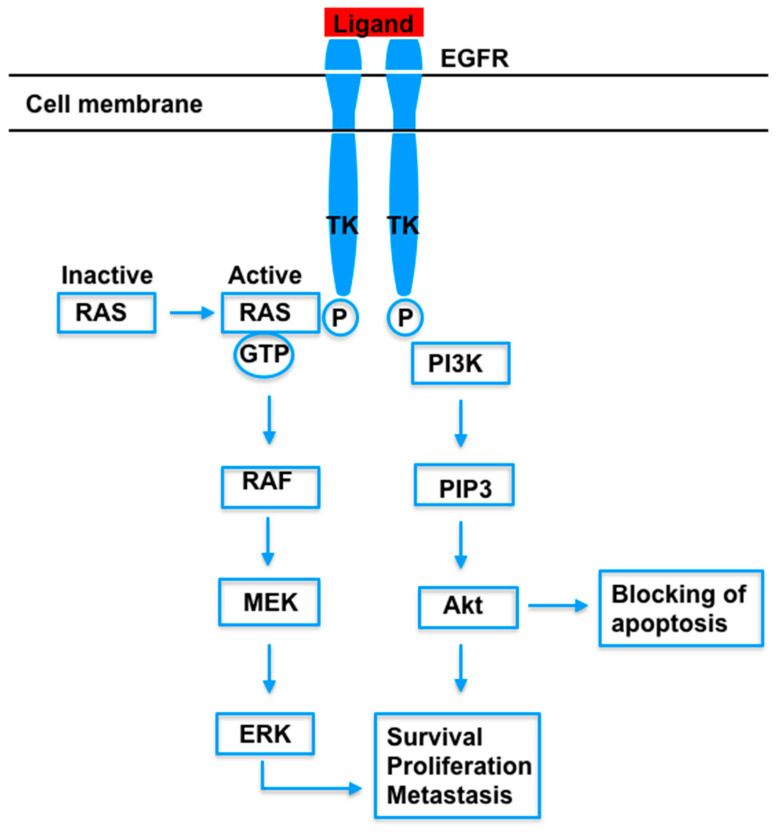
Steps of some signaling pathways activated by the binding of a ligand to EGFR. EGFR = endothelial growth factor receptor. TK = tyrosine kinase domain. P = phosphate. RAS = rat sarcoma protein. GTP = guanosine triphosphate. RAF = rapidly accelerated fibrosarcoma protein. MEK = mitogen-activated protein kinase. ERK = extracellular signal-regulated kinase. PI3K = phosphatidylinositol 3-kinase. PIP3 = phosphatidylinositol-3,4,5-trisphosphate kinase. Akt = protein kinase B.

The epidermal growth factor receptor (EGFR) gene, located on chromosome 7, is considered one of the driver genes that determine the carcinogenesis of NSCLC [33]. This gene leads to the production of a cell-surface, which possesses four extracellular and three intracellular domains, bound by a transmembrane sequence. The extracellular domains of the EGFR protein can bind epidermal growth factor molecules. This binding results in structural changes that lead to the dimerization of two EGFR proteins, which activate the intrinsic tyrosine kinase activity in the intracellular domain. The dimerized part can phosphorylate its intracellular C-terminal domains, which allows EGFR to interact with molecules that kickstart signaling pathways. The pathways activated by EGFR include MAPK (mitogen-activated protein kinase pathway), PI3K (phosphatidylinositol 3-kinase pathway), STAT3 (signal transducers and activators of transcription 3 pathway), and STAT5 (signal transducers and activators of transcription 5 pathway), which are involved in blocking apoptosis and stimulating cell survival, proliferation, and migration. Some of the steps of these pathways are shown in Figure 2 [34,35].

A meta-analysis from 2016 found that around 32.3% of NSCLC tumors harbor mutations in the EGFR gene. This mutation is more common in the female population (mutation rates are 19.7% higher than in males), Asians (rates are 52% higher than in North American populations), non-smokers (rates are 27.8% higher than in past or current smokers), and adenocarcinoma histology (rates are 26.3% higher than in other histologies) [36,37]. Around 90% of all EGFR mutations consist of either a guanine deletion in exon 19 (G719X) or substitution of leucine for arginine in exon 21 (L858R); these are called the “common mutations” [38]. Uncommon mutations occur in 10 to 18% of mutant tumors and include alterations such as insertions in exon 20 and point mutations in exon 18 (L861Q, S768I, G718X, amongst others) [39]. These mutations in the EGFR gene modify the structure of the tyrosine kinase domain. In many cases, the protein is activated, stimulating the MAPK, PI3K, and STAT pathways even in the absence of binding growth factors, consequently causing uncontrolled cell proliferation [40].

Anti-EGFR therapy consists of tyrosine kinase inhibitors (TKIs), target drugs that bind EGFR. First-generation drugs bind in a reversible manner to the ATP-binding site of EGFR and inhibit phosphorylation, blocking the signaling pathways associated with EGFR. Second-generation TKIs irreversibly block the tyrosine-kinase activity of EGFR by causing a covalent modification of this protein’s catalytic domain [41].

First-generation TKIs include erlotinib, gefitinib, and icotinib. Second-generation TKIs include afatinib and dacomitinib, which form irreversible bonds to the tyrosine kinase domains of wild-type and mutant EGFR, and also to other proteins of the same family (such as ERBB2—Erythroblastic Leukemia Viral Oncogene Homolog 2 protein, ERBB4—Erythroblastic Leukemia Viral Oncogene Homolog 4 protein) [42]. Various phase III clinical trials have shown the benefit of first and second-generation TKIs when compared to platinum-based chemotherapy in advanced NSCLC. The ENSURE trial enrolled two-hundred seventy-five patients who were randomized to receive either erlotinib or gemcitabine plus cisplatin. Progression-free survival was 11.0 versus 5.5 months (hazard ratio—HR = 0.34, 95% CI 0.22–0.51). The response rate in the erlotinib group was superior to the platin group (62.7% versus 33.6% respectively). The median overall survival was similar in the two groups [43]. The LUX-Lung 3 trial demonstrated superior progression-free survival in the afatinib group than in the cisplatin plus pemetrexed group (11.1 versus 6.9 months) and superior response rates (56% vs. 23%; *p* < 0.0001) [44].

The efficacy of first and second-generation TKIs depends on the mutation that EGFR harbors. Around 70% of all EGFR-mutated tumors respond clinically to TKIs, shrinking considerably in response to these drugs. The remaining 30% are intrinsically resistant to TKI drugs (which is referred to as de novo resistance) and do not respond to this treatment. This resistance is associated with EGFR exon 20 duplications or other mutations, such as PTEN (phosphatase and tensin homolog protein) and PIK3CA (phosphatidylinositol-4,5-bisphosphate 3-kinase catalytic subunit alpha) [45].

Despite these great results, first and second-generation TKIs have considerable toxicity. These drugs are more likely to cause side effects such as stomatitis, diarrhea, skin rash, and paronychia, requiring dose reduction in around 40% of these patients [46].

### 2.2. T790M Targeting

Despite the great clinical benefits of first and second-generation TKIs, almost all patients who undergo TKI treatment will develop resistance in a median of 9 to 13 months of treatment [47]. The clinical presentation of this acquired resistance is defined by a systemic progression of the disease for at least 30 days under continuous TKI treatment, preceded by a period of at least 6 months of clinical benefit [48]. The most common cause resistance, accounting for up to 60% of cases, is the substitution of methionine for threonine in position 790 of exon 20 (T790M). This change of amino acid leads to structural alterations where TKI drugs bind to EGFR. The side chain of the threonine molecule hinders the binding process of TKIs, leading to a resistance to these drugs [49].

To overcome T790M-mediated resistance, third-generation TKI drugs, such as osimertinib, mereletinib, and rociletinib were developed. These drugs bind irreversibly and with high potency to EGFR proteins with T790M mutations, leading to a powerful inhibition of cancer growth. The selectivity of third-generation TKIs for mutant EGFR proteins means that they spare wild-type EGFR, leading to fewer toxic side effects [50]. The phase III AURA study found that osimertinib increased PFS by 5.7 months and disease control rate (DCR) by 19% when compared to standard chemotherapy for NSCLC [51]. The phase III FLAURA study compared osimertinib to first-generation TKIs and found statistical gain in PFS, which extended from 10.2 to 18.9 months [52].

Another potential strategy to overcome T790M-mediated resistance is to cause the oxidation of the methionine residue of the mutated receptor, which leads to its degradation, and therefore decreases the proliferation pathways activated by EGFR. The oxidation can be achieved by the release of reactive oxygen species (ROS) in the NSCLC cells’ environment, through molecules such as sanguinarines. In the presence of ROS, the mutant EGFR is internalized and broken down by caspase enzymes. ROS also have a cytotoxic effect, leading to cell death [53].

Besides methionine oxidation, another promising strategy in fighting drug-resistant NSCLC is the use of polymers and nanoparticles to transport the target-therapy drugs directly into the tumor cells. The polymer is used to carry the drug, and the nanoparticle is specifically designed to bind to cell membrane proteins, which increases the penetration of the polymer into the cancerous cells. Recent studies have shown that a polymer carrying icotinib, ushered in by an amidoamine nanoparticle, has the ability to cause 87.56% tumor inhibition in mice models [54]. Another upcoming perspective in targeting resistance in NSCLC is the association of cytotoxic drugs with nanoparticles to increase the potency of this line of treatment. A 2019 study reported an association of 5-fluorouracil, an inhibitor of DNA synthesis, with Ag2S quantum dots, which are nanoparticles that emit light in the visible spectrum. The quantum dots increased the drug’s uptake by the cancer cells in the in vitro models reported in the study. Furthermore, while 5-fluorouracil showed a cell death rate of 20%, 5-fluorouracil combined with the quantum dots presented a cell death rate of 40% in cells with overexpressed EGFR [55].

### 2.3. ALK Inhibitors

Anaplastic lymphoma kinase (ALK) gene alterations are of great importance in NSCLC because they are target to medications and are present in approximately 5% of patients. These alterations include ALK gene rearrangement or fusion with echinoderm microtubule-associated protein-like 4 (EML4). Below are some of the current evidence for ALK inhibitors [56].

Crizotinib is an ALK inhibitor that was compared with standard chemotherapy as first-line therapy for advanced ALK-positive NSCLC, in an open-label phase 3 trial. The PFS was significantly longer in the Crizotinib arm compared to the chemotherapy arm (10.9 months vs. 7.0 months, *p* < 0.001); however, a superior median OS was not reached. The Crizotinib group reported adverse effects such as nausea, fatigue, vomiting, and decreased appetite [57]. Solomon et al. conducted the phase III PROFILE 1014 trial, which compared Crizotinib with chemotherapy as a first-line treatment for patients with ALK-positive NSCLC. Crizotinib was given at a dose of 250 mg twice daily. The standard group received IV Pemetrexed 500 mg/m^2^ + Cisplatin 75/m^2^ or Carboplatin every 3 weeks for a maximum of six cycles. The median OS was not reached for the Crizotinib group and was 47.5 months with conventional chemotherapy [58].

Alectinib is a highly selective ALK inhibitor. In a randomized, open-label, phase 3 trial, Peters et al. compared the effect of Alectinib (600 mg twice daily) with Crizotinib (250 mg twice daily) on ALK-positive NSCLC patients. The rate of investigator-assessed PFS was higher in the first group (68.4% vs. 48.7%, *p* < 0.001). Alectinib also demonstrated less grade 3 or 5 adverse events than Crizotinib (41% vs. 50%) [59]. A randomized, phase III trial, enrolled 303 chemotherapy-naïve patients to receive either Alectinib (600 mg twice daily) or Crizotinib (250 mg twice daily). The primary endpoint was investigator-assessed PFS and secondary endpoints were independent committee PFS, time to central nervous system (CNS) progression, ORR, and OS. During a median follow-up of 17.6 months (Crizotinib) and 18.6 months (Alectinib), disease progression occurred in 41% in the Alectinib group and 68% in the Crizotinib group. Response occurred in 82.9% of patients in the Alectinib group and 75.5% in the Crizotinib group. Grade 3 to 5 adverse events were less frequent with Alectinib (41 vs. 50% Crizotinib) [60].

Ceritinib, a next-generation ALK inhibitor, assessed efficacy and security in the front line setting in the ASCEND-4 trial. In this open-label, randomized, phase III trial, ceritinib (750 mg daily) was compared with platinum-based chemotherapy (Cisplatin 75 mg/m^2^ or Carboplatin AUC 5-6 plus Pemetrexed 500 mg/m^2^) every three weeks for four cycles followed by maintenance Pemetrexed. The primary endpoint was reached, with median PFS survival of 16.6 months in the Ceritinib group and 8.1 months in the chemotherapy group (*p* < 0.00001). The most common adverse events were diarrhea, vomiting, and increasing in alanine aminotransferase in the Ceritinib group. Ceritinib showed statistically significant and clinically meaningful improvement in PFS versus chemotherapy in the first-line setting [61].

Brigatinib is a next-generation potent ALK inhibitor that has demonstrated efficacy in patients whose treatment failed with Crizotinib. However, the efficacy in the first-line setting was unclear. Camidge et al. conducted an open-label, phase 3 trial with Brigatinib (180 mg once daily) versus Crizotinib (250 mg twice daily) for those who had not previously received an ALK inhibitor. The PFS was a primary endpoint and was higher in the Brigatinib arm (67% vs. 43% at 12 months follow up, with HR for progression or death, 0.49; *p* < 0.001), with no new concerns about safety [62].

Regarding adverse events, a linkage was made between ALK inhibitors, such as Alectinib, Ceritinib, Crizotinib, and Brigatinib to pneumonitis. The overall incidence of ALK inhibitor-related pneumonitis was 2.14% in patients with advanced NSCLC [63]. ALK inhibitors have also been associated with liver toxicities, raising the levels of alanine transaminase (ALT) and aspartate aminotransferase (AST) in 26.0% and 23.2% of patients, respectively [64].

### 2.4. c-MET

The c-MET (mesenchymal epithelial transition) protein is another key factor in the progression of NSCLC, making this a promising target for new cancer therapies. c-MET is a tyrosine kinase receptor, activated by the binding of a hepatocyte growth factor (HGF) molecule in the extracellular domains of the protein. HGF causes the dimerization of two adjacent c-MET receptors, which alters the tridimensional structure of the protein in a manner that allows the auto-phosphorylation and subsequent activation of the tyrosine-kinase domains in the extracellular part of c-MET. The active catalytic domains of c-MET will lead to the phosphorylation of messengers involved in pathways such as MAPK, PI3K, and STAT3. As mentioned before, these are pathways related to cell proliferation, invasion, and undifferentiation, and therefore contribute to oncogenesis [65,66].

Alterations in the c-MET gene, located in chromosome 7q21-31, have been widely reported in NSCLC tissues. The most frequent c-MET gene alteration is overexpression, which was identified in up to 72% of NSCLC tumors, through identification of abnormally high levels of c-MET RNA in the patients’ bloodstream. Skipping mutation in exon 14 of c-MET has been reported in around 4% of NSCLC cases. These mutations cause lower rates of c-MET ubiquitination and degradation, therefore leading to amplified proliferation pathways and more aggressive tumor characteristics [66,67]. The amplification of the c-MET gene, reported in up to 20% of patients, has been linked to an acquired resistance to EGFR TKI drugs. In the presence of EGFR inhibitors, the tumor cells respond with MET amplification, which allows the maintenance of hyperactivated cancer pathways, leading to tumor progression despite EGFR TKI treatment. A recent Chinese study showed that patients with acquired resistance to Osimertinib via MET amplification had shorted PFS compared with patients who did not harbor this MET alteration (15.6 months versus 30.7 months, respectively) [68,69].

Therefore, drugs targeting the mechanisms involved in c-MET activation are important tools in the fight against NSCLC. These drugs include inhibitors of the MET tyrosine-kinase activity (Crizotinib, Tepotinib, Capmatinib), monovalent monoclonal antibodies that target MET (Onartuzumab) or HGF (Rilotumumab) [70].

MET inhibitors bind to the intracellular catalytic domain of MET and deter the phosphorylation of second messengers in the MAPK, PI3K, and STAT3 pathways, decreasing cell proliferation and invasion. The AcSé phase II trial testes Crizotinib and found an ORR of 36% for patients with MET mutations, and one of 32% for patients with MET amplification (copy number superior to 6) [71]. The METROS phase II trial found a median ORR of 27% and a PFS of 4.4 months for patients with MET mutations or amplifications treated with Crizotinib [72]. Capmatinib showed more promising results. It is highly selective to MET and has a high inhibition potency, being up to 30 times more potent than Crizotinib, and recedes the EGFR TKI resistance in tumor cells. Capmatinib mainly targets amplified and exon 14 mutated MET. The GEOMETRY Mono-1 trial studied the effect of Capmatinib in advanced NSCLC and found an ORR of 71.4% and a duration of response of 8.41 months (immature at the cut-off rate) for this drug. The study also found that the PFS was higher by almost 4 months in previously untreated patients when compared to previously treated patients [73]. Another study which associated Capmatinib with Gefitinib found a DCR of 73% for the combination of both drugs, and a median PFD of around 5.4 months for the patients who underwent this line of treatment [74].

In the phase II JO28638 trial, which analyzed patients with MET amplification and EGFR mutation, Onartuzumab showed an ORR of 68.9% and a PFS of 8.5 months when compared to Erlotinib [75]. Another study combined Onartuzumab with Bevacizumab and Pemetrexed-based chemotherapy and found that Onartuzumab did not provide benefits when added to these therapies, and only increased the rates of adverse effects such as peripheral edema and thromboembolism [76]. The anti-HGF antibody Rilotumumab showed a DCR of 60% and a PFS of 2.6 months [77].

### 2.5. Immunotherapy

Programmed death 1 (PD-1) is a key immune checkpoint receptor present on the surface of activated T, B, and NK cells. As such, PD-1 along with its ligand (PD-L1) plays an important role in tumor immune escape, inducing T-cell apoptosis, or exhaustion. PD-1 and PD-L1 are immunotherapy targets. Their inhibition has been used to treat patients with NSCLC without EGFR or ALK mutations. Some examples of immunotherapy agents are Pembrolizumab, Nivolumab, and Atezolizumab approved as second-line therapies or in combination with chemotherapy in the first-line [78]. 

A double-blind, phase 3 trial (KEYNOTE 189) compared Pemetrexed and platinum-based chemotherapy and Pembrolizumab (200 mg) or Placebo. Pembrolizumab significantly improved the OS in combination with chemotherapy at 12 months (69.2% vs. 49.4%, *p* < 0.001); this benefit was seen across all PD-L1 subgroups; however adverse effects grade 3 or higher were a bit higher in the Pembrolizumab-combination group, compared to the placebo group (67.2% vs. 65.8%) [79]. The KEYNOTE 024 study randomly assigned 305 previously untreated patients and compared pembrolizumab versus standard therapy (platinum-based chemotherapy at the investigator´s choice). PD-L1 more than 50% was required. Median progression-free survival was 10.3 months in the pembrolizumab group vs. 6.0 months in the chemotherapy group; this difference was statistically significant. Adverse events were lower in the pembrolizumab group [80].

Nivolumab was assessed at the second line by the CheckMate 017 and CheckMate 057 trials. Checkmate 057 evaluated patients with non-squamous NSCLC after progression during or after platinum chemotherapy. Nivolumab was administered at 3 mg/kg every 2 weeks, while the Docetaxel dose was 75 mg/m^2^ every 3 weeks. After 40.3 months’ minimum follow-up, Nivolumab achieved higher OS at 18 months (39%) compared to Docetaxel (23%). This efficacy was independent of PD-L1 levels. No new safety concerns were identified [81].

Hellmann et al. conducted an open-label, phase 3 trial comparing the efficacy and safety of Nivolumab plus Ipilimumab, Nivolumab alone or chemotherapy, in patients with stage IV or recurrent NSCLC. Ipilimumab is a fully human anti-cytotoxic T-lymphocyte antigen 4 (CTLA-4) antibody that has already been associated with Nivolumab to treat patients with melanoma or renal-cell carcinoma. In the group of patients with PD-L1 expression of 1% or more, median OS was 17.1 months (95% CI, 15.0 to 20.1) for the combined arm and 14.9 months (95% CI, 12.7 to 16.7) for chemotherapy (*p* = 0.007). Median DOR also showed benefit in the combined arm (23.2 months) rather than chemotherapy (6.2 months). As for the patients with a PD-L1 expression level <1%, OS was of 17.2 months (95% CI, 12.8 to 22.0) for the combined arm and 12.2 months (95% CI, 9.2 to 14.3) for those treated with chemotherapy. Considering the entire study population, the incidence of grade 3 or 4 treatment-related adverse events was higher with chemotherapy (36.0%) than nivolumab plus ipilimumab (32.8%), and no new safety concerns were found [82].

Atezolizumab was evaluated in an open-label, phase 3 trial by Socinski et al. Patients were randomly assigned to receive either Atezolizumab + Carboplatin + Paclitaxel (ACP), Bevacizumab + Carboplatin + Paclitaxel (BCP), or Atezolizumab + BCP (ABCP), followed by maintenance therapy with Atezolizumab, Bevacizumab, or both. In the entire intention-to-treat population, PFS was longer in the ABCP group than in the BCP one (8.3 months vs. 6.8 months, *p* < 0.001). The most common grade 3 or 4 treatment-related adverse effects were neutropenia, decreased neutrophil count, febrile neutropenia, and hypertension [83].

The OAK trial is a phase 3, open-label, multicentric, and randomized controlled trial that compared Atezolizumab (1200 mg) versus Docetaxel (75 mg/m^2^) in patients with previously treated NSCLC. Atezolizumab significantly increased OS in the intention-to-treat and PD-L1-expression population (at least 1% PD-L1 on tumor cells or tumor-infiltrating immune cells), 13.8 months for Atezolizumab (95% CI, 11.8 to 15.7) and 9.6 months (95% CI, 8.6 to 11.2) for those who received Docetaxel (HR 0.73, *p* = 0.0003). As for patients with low or undetectable PD-L1 (TC0 and IC0), Atezolizumab also improved OS (12.6 months versus 8.9 months, 95% CI 0.59 to 0.96, HR 0.75). OS was improved similarly in patients with squamous or non-squamous histology. Docetaxel was associated with a higher rate of treatment-related grade 3 or 4 adverse events (43% of 578 patients) and had one treatment-related death from a respiratory tract infection, while 15% of the Atezolizumab group (609 patients) reported grade 3 or 4 adverse events [84].

### 2.6. Systemic Therapy

Systemic therapy consists of chemotherapy, targeted therapy, or immunotherapy, each one with its own indications. For instance, patients with NSCLC harboring sensitizing EGFR-mutations documented before the beginning of first-line therapy should receive Erlotinib, Gefitinib, or Afatinib instead of standard chemotherapy. Crizotinib can be used to treat patients with advanced NSCLC who have ALK gene rearrangements or ROS1 rearrangements; however, should be discontinued if the patient presents life-threatening pneumonitis. It is also recommended as subsequent therapy in those patients with ALK-positive disease. Alectinib was shown to be active in patients with ALK rearrangements who had progressed on Crizotinib. As for chemotherapy, a phase III randomized trial presented Ramucirumab/Docetaxel as an option for subsequent therapy for all histologic subtypes of NSCLC. Other meaningful chemotherapy agents are Vinorelbine, Ifosfamide, and Pemetrexed. The last one is sometimes associated with Cisplatin or Carboplatin [85].

## 3. Discussion

EGFR resistance target-therapy for advanced NSCLC is one of the biggest challenges to be overcome. Around 15% of cases of acquired resistance to first and second-generation TKIs remain unexplained, which means the molecular mechanisms behind these clinical expressions are still unknown [49]. Furthermore, while resistance via T790M mutation can be overcome with the use of third-generation TKIs (i.e., osimertinib), most patients will develop resistance even to these third-generation drugs. FLAURA trial established Osimertinib as a standard of care in the frontline, whereas impressive results over Crizotinib have been demonstrated, with PFS 18.9 months vs. 10.2 months, HR 0.46; 95% confidence interval (CI), 0.37 to 0.57; *p* < 0.001); in a follow up at 3 years, 79 of 279 patients (28%) in the Osimertinib group and 26 of 277 (9%) in the Crizotinib were alive [86]. This scenario raised the concern of which is the best choice when resistance to a third-generation TKI is acquired. Mutations such as C797S, L718Q, and L844V in the tyrosine kinase domain of EGFR have all been associated with resistance to one or more third-generation TKIs. C797S-generated resistance possibly will be overcome by a fourth-generation inhibitor, EAI045. This drug achieved great tumor regression when administered with cetuximab in mouse models carrying L858R, T790M, and C797S mutations. Further studies are necessary to determine its role in EGFR inhibition and possible effects in humans [87]. Besides, resistance to all types of TKIs can be acquired by activation of alternative pathways in the proliferation of cancer cells, which can be achieved with BRAF (murine sarcoma viral oncogene homolog B1) mutation and MET (hepatocyte growth factor receptor) or HER2 (human epidermal growth factor receptor 2) amplification [49]. This opens up the possibility of combining anti-EGFR drugs with other target therapies according to each tumor’s molecular characterization, which should become progressively more common in the era of personalized medicine.

Anti-ALK therapy is one of the backbones in the fight against NSCLC; Crizotinib led to important progress in this scenario and is considered more efficient and less toxic than standard chemotherapy regimens. Alectinib demonstrated impressive results in the ALEX trial published in 2017, is considered the standard of care, offered in the frontline setting to patients harboring ALK gene fusion [56]. Ceritinib has also significantly improved endpoints when compared to chemotherapy with pemetrexed, despite a lower response rate in CNS when cross-trial compared to Alectinib and Brigatinib [58]. This third-generation drug showed positive results in the phase III trial ALTA-1, with superior CNS responses when compared to Crizotinib [59]. Nevertheless, when thinking from the health system perspective, more evidence from the pharmacoeconomic field is lacking, considering the high cost and the need for smart and concise decisions, especially when we think of limited resources to be shared with several diseases and conditions.

Pembrolizumab was the backbone of immunotherapy in lung cancer. The revolutionary trial, KEYNOTE 024, changed the future of chemotherapy in lung cancer, setting back cytotoxic agents to later lines of treatment and approved Pembrolizumab replacing chemotherapy for PD-L1 > 50% patients [64]. Furthermore, Pembrolizumab has been approved in combination with chemotherapy in the phase III trial Keynote 0189, and in the phase II trial Keynote 021 for patients expressing PD-L1 from 1 to 50% [88]. The addition of pembrolizumab also improved outcomes in squamous cell cancers, without adding substantial toxicity.

Nivolumab significantly improved OS in the second line against docetaxel, setting back again chemo agents. The combination of Nivolumab and Ipilimumab has demonstrated a superior OS compared with chemotherapy in the Checkmate 227 trial. Concerning adverse events has risen, especially when the combination is used; however, in this trial, grade 3 or 4 events occurred in about 33% of the patients using immunotherapy versus 36% with chemotherapy [66].

Atezolizumab improved PFS as part of combined treatment and presented fewer adverse effects than Docetaxel in patients with previously treated NSCLC [79]. It also has been compared to chemotherapy alone and in combination in the frontline at IMPOWER 131 trial. In a median follow-up of 17 months, those assigned to atezolizumab and chemotherapy experienced an improved PFS (6.3 versus 5.6 months; HR 0.7, 95% CI 0.60–0.85) relative to those receiving chemotherapy, with greatest benefits for those with high PD-L1 expression (≥50 percent) and no benefit for those with PD-L1-negative tumors [89].

In the 2017 NCCN guidelines, metastatic NSCLC is treated according to its histologic subtype and biomarker testing. Squamous cell carcinomas should be tested for EGFR (if positive, first-line therapy should consist of Erlotinib, Afatinib or Gefitinib), ALK (if positive, consider Crizotinib or Ceritinib), ROS1 (if positive, consider Crizotinib) and PD-L1 (if PD-L1 positive with the aforementioned markers either negative or of unknown status, consider systemic immune checkpoint inhibitors, Nivolumab, Pembrolizumab, Atezolizumab or systemic therapy with Docetaxel, Pemetrexed, Gemcitabine or Ramucirumab + Docetaxel). As for adenocarcinomas, large cell carcinomas and not otherwise specified NSCLC the same biomarkers should be evaluated; however, first-line therapy differs from squamous NSCLC when PD-L1 positive and EGFR- ALK and ROS-1 negative or of unknown status (Nivolumab, Pembrolizumab, Atezolizumab or systemic therapy with Docetaxel, Gemcitabine or Ramucirumab + Docetaxel). Palliative care should be considered according to each case [85].

Other drugs are still being tested in major clinical trials, improving scientific knowledge, and opening new possibilities for treating NSCLC. One example is the Checkmate 9LA trial, comparing the combination of Nivolumab and Ipilimumab with chemotherapy or chemotherapy alone, which will soon be published. The PACIFIC trial assesses the use of Durvalumab after chemotherapy in stage III NSCLC, with significant improvements in OS [90] The soon to be published ADAURA trial obtained unprecedented results with osimertinib in EGFR-positive NSCLC. Other current trials found in the National Institute of Health’s database can be seen in Table 1 [91].

Recent publications also added important information to the state of art regarding NSCLC treatment. Rossi [92]. reviewed registrative trials concerning advanced non-squamous non-oncogene-addicted NSCLC and concluded that those with PD-L1 expression <50% benefit from first-line combinations of Pembrolizumab or Atezolizumab with chemotherapy, or second-line therapy with antiangiogenic agents plus Docetaxel (Table 2). Wang et al. [93] conducted a retrospective analysis of both metastatic melanoma (*n* = 90) and metastatic NSCLC patients (*n* = 37) treated with immune checkpoint inhibitors, with or without concurrent cyclo-oxygenase inhibitor treatment, between 2011 and 2019. The drug combination showed benefit both in time-to-progression and ORR at 6 months for metastatic melanoma and NSCLC patients. A phase 1 study by Creelan et al. [94] analyzed the combination of Gefitinib and Durvalumab on 56 EGFR TKI-naïve patients with EGFR mutation-positive locally advanced or metastatic NSCLC. The combination arm of the trial presented higher toxicity than either drug alone, therefore physicians should avoid the combination of PD-L1 inhibitors and Gefitinib in TKI-naïve patients with EGFR mutated NSCLC.

## Figures and Tables

**Table 1:** Abbreviations used throughout this review.

Abbreviation	Meaning	First Appearance
LC	Lung Cancer	37
WHO	World Health Organization	37
NSCLC	Non-Small Cell Lung Cancer	43
OS	Overall Survival	95
RR	Response Rate	106
VEGFR	Vascular Endothelial Growth Factor Receptor	126
PFS	Progression-Free Survival	142
ORR	Overall Response Rate	150
EGFR	Epidermal Growth Factor Receptor	164
MAPK	Mitogen-Activated Protein Kinase Pathway	171
PI3K	Phosphatidylinositol 3-Kinase Pathway	172
TKI	Tyrosine Kinase Inhibitor	187
T790M	Substitution of Methionine for Threonine in the Position 790 of Exon 20	227
DCR	Disease Control Rate	235
ROS	Reactive Oxygen Species	241
ALK	Anaplastic Lymphoma Kinase	259
CNS	Central Nervous System	278
MET	Mesenchymal Epithelial Transition	305
PD-1	Programmed Death 1	353
PD-L1	Programmed Death Ligand 1	354

**Table 2:** Some of the current clinical trials on NSCLC treatment MET inhibitors [69]. R = recruiting; NYR = not yet recruiting; ANR = active, not recruiting. CCC = Comprehensive Care Center. Enrollment is presented as an estimated number of participants. Start and end dates account for the estimated study start or conclusion date respectively.

Study Title	Status	Condition	Intervention	Phase	Location	Enrollment	Start	End
Nivolumab Plus Ramucirumab in Patients with Recurrent, Advanced, Metastatic NSCLC	NYR	NSCLC	Nivolumab Ramucirumab	2	Fox Chase Cancer Center (Philadelphia, PA, USA)	117	June 2020	April 2024
Efficacy of Epidermal Growth Factor Receptor Tyrosine Kinase Inhibitor Combined with Early Stereotactic Body Radiation Therapy to the Primary Tumor in Advanced Non-small Cell Lung Cancer	NYR	Advanced NSCLC	EGFR-TKI Stereotactic Body Radiation Therapy	3	Taizhou Hospital, WMU (Taizhou, Zhejiang, China)	300	June 2019	June 2022
A Study Evaluating Platinum-Pemetrexed-Atezolizumab (+/-Bevacizumab) for Patients with Stage IIIB/IV Non-squamous Non-small Cell Lung Cancer with EGFR Mutations, ALK Rearrangement or ROS1 Fusion Progressing After Targeted Therapies (GFPC 06-2018)	R	NSCLC Stage IIIB NSCLC Stage IV EGFR Mutation ROS1 Mutation ALK Rearrangement	Carboplatin Pemetrexed Atezolizumab Bevacizumab	2	CHU Angers, France Centre Hospitalier (Annecy, France) CHU-Hôpital MORVAN (Brest, France) and 20 more	149	September 2019	June 2023
First-line Treatment with Osimertinib in EGFR-mutated Non-small Cell Lung Cancer (FIOL)	R	Lung Cancer	Osimertinib	2	Aarhus University Hospital (Aarhus, Denmark) Herlev Hospital (Copenhagen, Denmark) Rigshospitalet (Copenhagen, Denmark) and 7 more	100	December 2018	March 2023
Targeted Treatment for ALK Positive Patients Who Have Previously Been Treated for Non-squamous Non-small Cell Lung Cancer	R	Non-Squamous NSCLC Stage IV AJCC v8 Stage IVA AJCC v8 Stage IVB AJCC v8	Alectinib Brigatinib Carboplatin Ceritinib Cisplatin Crizotinib Ensartinib Lorlatinib Pemetrexed	2	CTCA at Western Regional Medical Center (Goodyear, AZ, USA) Mayo Clinic Hospital (Phoenix, AZ, USA) Mayo Clinic (Scottsdale, AZ, USA) and 453 more	660	April 2019	December 2025
Osimertinib In EGFR Mutant Lung Cancer	R	NSCLC	Osimertinib	2	Beth Israel Deaconess Medical Center (Boston, MA, USA) Dana-Farber Cancer Institute (Boston, MA, USA)	30	August 2018	March 2023
A Randomized Two Arm Phase II Trial of Pembrolizumab Alone or Sequentially Following Single Fraction Non-Ablative Radiation to One of the Target Lesions, in Previously Treated Patients with Stage IV NSCLC	R	Stage IV NSCLC	Pembrolizumab Single Fraction Radiation Therapy	2	Cleveland Clinic Taussig Cancer Institute, Case CCC (Cleveland, OH, USA)	48	May 2017	December 2020
A Study to Evaluate Efficacy and Safety of Multiple Targeted Therapies as Treatments for Participants with Non-Small Cell Lung Cancer (NSCLC) (B-FAST)	R	NSCLC	Alectinib Atezolizumab Pemetrexed Cisplatin Carboplatin Gemcitabine Entrectinib Cobimetinib Vemurafenib	2/3	University of California (La Jolla, San Diego, CA, USA) UC Davis CCC (Sacramento, CA, USA) Rocky Mountain Cancer Center (Denver, CO, USA) and 178 more	660	September 2017	September 2021
Study of TQ-B3139 Versus Crizotinib in the First Line Treatment of Subjects with Anaplastic Lymphoma Kinase (ALK) Positive Non-Small Cell Lung Cancer (NSCLC)	R	ALK-positive NSCLC	TQ-B3139 Crizotinib	3	Sun Yat-Sen University Cancer Center (Guangzhou, Guangdong, China)	260	August 2019	April 2022
A Study of Osimertinib With or Without Chemotherapy as 1st Line Treatment in Patients with Mutated Epidermal Growth Factor Receptor Non-Small Cell Lung Cancer (FLAURA2)	R	NSCLC	Osimertinib Pemetrexed Carboplatin Cisplatin	3	Bellflower (CA, USA) La Jolla (CA, USA) Oxnard (CA, USA) and 107 more	586	July 2019	March 2026
A Global Study to Assess the Effects of MEDI4736 Following Concurrent Chemoradiation in Patients with Stage III Unresectable Non-Small Cell Lung Cancer (PACIFIC)	ANR	NSCLC	MEDI4736	3	Chandler, (AZ, USA) Goodyear (AZ, USA) Fayetteville, (AR, USA) and 230 more	713	May 2014	March 2021
